# The Role of CD38 on the Function of Regulatory B Cells in a Murine Model of Lupus

**DOI:** 10.3390/ijms19102906

**Published:** 2018-09-25

**Authors:** Brianna Burlock, Gabrielle Richardson, Sonia García-Rodríguez, Salvador Guerrero, Mercedes Zubiaur, Jaime Sancho

**Affiliations:** 1 Department of Cellular Biology and Immunology, Instituto de Parasitología y Biomedicina López Neyra, Consejo Superior de Investigaciones Científicas (IPBLN-CSIC), 18016 Granada, Spain; bburlock@scmail.spelman.edu (B.B.); richardsg1126@gmail.com (G.R.); garciarodriguez.sonia@gmail.com (S.G.-R.); mzubiaur@ipb.csic.es (M.Z.); 2 Flow-Cytometry Unit, IPBLN-CSIC, 18016 Granada, Spain; salvaguerrero@ipb.csic.es

**Keywords:** Bregs, CD38, CD1d^hi^, lupus, IL-10, autoimmunity, inflammation

## Abstract

Previous work from our group has shown that *Cd38^−/−^* mice develop a milder pristane-induced lupus disease than WT or *Art2^−/−^* counterparts, demonstrating a new role for CD38 in promoting aberrant inflammation and lupus-like autoimmunity via a Transient Receptor Potential Melastatin 2 (TRPM2)-dependent apoptosis-driven mechanism. In this study we asked whether CD38 may play a role in the expression and function of regulatory B cells (IL-10-producing B cells or B10 cells). In pristane-treated mice the frequency of spleen CD19^+^CD1d^hi^CD5^+^ B cells, which are highly enriched in B10 cells, was significantly increased in *Cd38^−/−^* splenocytes compared to WT, while the frequency of peritoneal plasmacytoid dendritic cells (pDCs), which are major type I Interferon (IFN) producers, was greatly diminished. The low proportion of pDCs correlated with lower amounts of IFN-α in the peritoneal lavage fluids of the *Cd38^−/−^* mice than of WT and *Art2^−/−^* mice. Functional ex vivo assays showed increased frequencies of IL-10-producing B cells in *Cd38^−/−^* splenocytes than in WT upon stimulation with an agonist anti-CD40 mAb. Overall these results strongly suggest that *Cd38^−/−^* mice are better suited than WT mice to generate and expand regulatory B10 cells following the appropriate stimulation.

## 1. Introduction

CD38 is a transmembrane glycoprotein with receptor-mediated signaling capabilities and is an enzyme that catalyzes nicotinamide adenine dinucleotide (NAD^+^) or nicotinamide adenine dinucleotide phosphate (NADP^+^) to produce molecules involved in Ca^2+^ messenger signaling (cyclic ADPR (cADPR), adenosine diphosphate ribose (ADPR), and nicotinic acid adenine dinucleotide phosphate (NAADP). Previous studies of our group in Systemic Lupus Erythematosus (SLE) patients show increased CD38 surface expression in T cells and low levels of anti-CD38 autoantibodies in clinically active patients. Furthermore, increased CD38 expression in SLE B cells correlated exclusively with plasma levels of IL-10 [1]. Other results from our group show that *IL-10* gene expression in SLE patients correlates with *CD38* gene expression; moreover, *CD38* expression correlates with *AKT* serine/threonine kinase 1 (*AKT1*) expression [2], and in human T and B cells CD38 receptor stimulation leads to Protein Kinase B (PKB)/AKT and Extracellular signal-Regulated protein Kinase (ERK) activation [3]. In a kinase-dead mutant of PI3Kp110δ mice, Patton et al. [4] reported that PI3Kp110δ kinase regulates expression of CD38 on regulatory T cells (Treg). These studies, combined with the observations that continuous administration of anti-IL-10 demonstrated a delay in onset of autoimmunity in NZB/W F_1_ mice [5], and anti-IL-10 mAb administered to six steroid-dependent patients with SLE was shown to have a beneficial effect on disease activity [6], suggest that this cytokine may promote disease. Other studies in IL-10 gene-deficient (IL-10^−/−^) MRL-*Fas^lpr^* (MRL-*Fas^lpr^* IL-10^−/−^) mice, however, suggest that IL-10 may play a suppressive role in lupus [7]. As suggested by others, these contradictory findings are most likely explained by the fact that multiple cell types are capable of producing IL-10, including B cells. Therefore, the positive and negative regulatory roles of IL-10 are likely to differ depending on the cell source of IL-10, as well as the timing of its production, duration, and levels of IL-10 expression [8].

Furthermore, Blair et al. [9] documented that human CD19^+^CD24^hi^CD38^hi^ B cells exhibit regulatory capacity in healthy individuals, while the same B cells from SLE patients produced less IL-10 and lacked the suppressive capacity. Our data showed an increase in *FOXP3*-(Treg) gene expression in SLE patients that positively correlated with *IL-10* gene expression [2]. Mouse regulatory B cells (IL-10-producing B cells or B10 cells) control T-cell autoimmunity through IL-21-dependent cognate interactions [10,11]. B10 cells are highly enriched in the spleen within the CD1d^hi^CD5^+^ B cell subset [12,13]. Prophylactic B cell depletion by repeated CD20 mAb treatments prolonged survival during pristane-accelerated lupus in NZB/W F_1_ mice, and also delayed spontaneous disease in NZB/W F_1_ mice. In contrast, B cell depletion initiated in 4-week-old mice hastened disease onset, which paralleled depletion of the B10 cells [14]. Note that the pathologic manifestations of nephritis appear significantly earlier, and survival is significantly reduced in NZB/W F_1_ mice that lack B10 cells because of constitutive CD19-deficiency [8]. In this study, CD19 deficiency led to lower serum IL-10 levels in NZB/W mice throughout the disease course. The transfer of splenic CD1d^hi^CD5^+^ B cells from wild type NZB/W F_1_ mice into CD19^−/−^ NZB/W F_1_ recipients significantly prolongs their survival [8]. Thus, B10 cell IL-10 production is but one component of a complex regulatory network that balances protective and pathogenic immune responses [15].

IL-10 seems to be involved in inhibiting some of the clinical/pathologic manifestations of pristane-induced lupus such as diffuse alveolar hemorrhage (DAH) [16]. Although the mechanism is still not fully understood, it seems that IL-10 protects against pristane-induced lung injury by interacting with IL-10R on alveolar macrophages or bone marrow-derived cells [16]. *Cd38^−/−^* mice develop a milder pristane-induced lupus disease than WT and *Art2^−/−^* mice [17]. Our data demonstrate that CD38 promotes pristane-induced chronic inflammation and increases susceptibility to experimental lupus by an apoptosis-driven and Transient Receptor Potential Melastatin 2 (TRPM2)-dependent mechanism [17]. On the other hand, NAD-induced cell death (NICD), which acts through the mono-ADP-ribosyltransferase 2(ART2)-P2X purinoreceptor 7 (P2X7) pathway [18,19], is regulated by CD38. Indeed, lack of CD38 in ART2^+^ T cells results in increased NICD, which correlates with a significant reduction in Tregs and immunoregulatory natural killer T (iNKT) cells, even under steady-state conditions [20]. Depending on the involved apoptotic T-cell subset, enhanced ART2 activity could result in immunosuppression or autoimmunity. For that reason, we have reported that lack of CD38 in a B6 genetic background ameliorates autoimmunity in the collagen-induced arthritis model due to decreased iNKT cells in secondary lymphoid organs that were unable to boost a Type 1 helper T cell (Th1) response [21]. Note that IL-10-producing NKT (NKT10) cells that resemble type 1 regulatory T cells have also been characterized [22]. Through the production of IL-10, αGalCer-pretreated iNKT cells impaired antitumor responses and reduced disease in experimental autoimmune encephalomyelitis, a mouse model of autoimmune disease [22].

We asked whether CD38 may play a role in Breg expression and function. To answer this question we investigated whether there were differences in Breg expression and function between WT and CD38-deficient mice in naïve mice. Also, we provide data on the frequencies of the CD1d^hi^CD5^+^ B cell subset, plasmacytoid dendritic cells (pDCs), and peritoneal levels of IFN-α in the pristane-induced lupus disease model.

## 2. Results

### 2.1. Similar Proportion of Splenic CD1d^hi^CD5^+^ B Cells in Naïve Cd38^−/−^ and WT Mice

**Figure 1:**
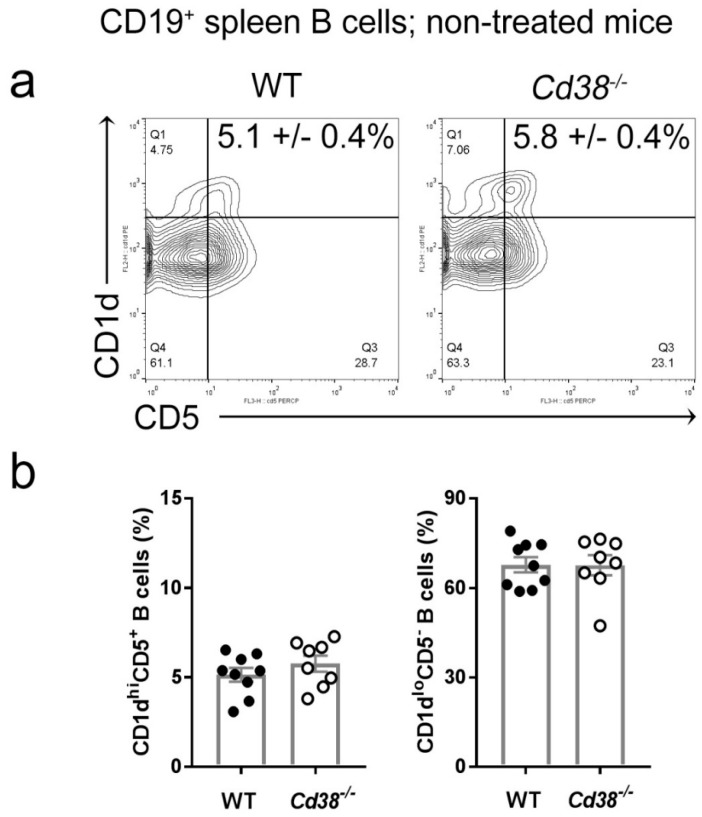
Similar proportion of splenic CD1d^hi^CD5^+^ B cells in naïve *Cd38^−/−^* and WT mice. Spleen cells were stained for cell surface CD1d, CD5, CD38, and CD19 as described in Material and Methods. Gating strategy is shown in Figure S1. (**a**) Representative contour-plots of CD1d and CD5 expression by CD19^+^ B cells from WT and *Cd38^−/−^* mice. Q1–Q4 quadrants show the frequencies of the four major B cell subsets. The mean frequencies ± SEM of the CD1d^hi^CD5^+^ cells are highlighted. (**b**) Frequencies ± SEM of the CD1d^hi^CD5^+^ (left) and CD1d^lo^CD5^−^ (right) B cells. Bars represent the mean frequencies and dots represent the individual values per mouse. Graph summarizes the results from nine independent experiments with a total of nine WT mice and eight *Cd38^−/−^* mice.

Since spleen regulatory B10 cells are highly enriched within the CD1d^hi^CD5^+^ B cell subset [12], we first assessed the frequency of these cells in naïve *Cd38^−/−^* and WT mice. As shown in Figure 1, naïve *Cd38^−/−^* mice showed a similar proportion of CD1d^hi^CD5^+^ B cells than in WT mice.

### 2.2. Increased Surface Expression of CD38 in WT CD1d^hi^CD5^+^ B Cell Subset

**Figure 2:**
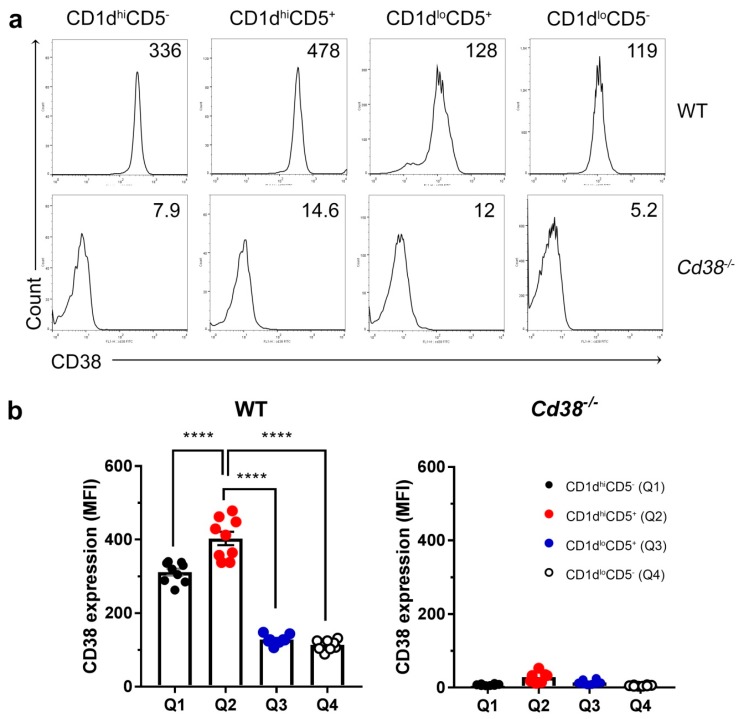
Increased surface expression of CD38 in WT CD1d^hi^CD5^+^ B cells. (**a**) Histograms represent CD38 expression determined by flow cytometry in each gated B cell subset from Figure 1a. Numbers within each panel represent the mean fluorescence intensities (MFI) for CD38 of a representative experiment performed in parallel in a naïve WT (upper panels) and in a naïve *Cd38^−/−^* mouse. (**b**) Mean ± SEM of MFIs for CD38 expression in the four B cell subsets from spleens of WT and *Cd38^−/−^* mice. Graph summarizes the results from nine independent experiments with a total of nine WT mice and eight *Cd38^−/−^* mice. **** *p* < 0.0001; Ordinary One-Way ANOVA test corrected using Tukey’s multiple comparisons test.

Increased CD38 surface expression is a hallmark of regulatory B cells in both human and mice [9,23]. Therefore, we tested CD38 surface expression in the four major B cells subsets (CD19^+^ cells) defined by the CD1d and CD5 markers. As shown in Figure 2, CD38 expression was significantly higher in the CD1d^hi^ subsets, either CD5^+^ or CD5^−^ than in the other two subsets. Note that the highest CD38 expression resides in the CD1d^hi^CD5^+^ subset; as these differences are statistically significant they confirm and extend previous results obtained by others [23].

### 2.3. Increased Frequency of CD1d^hi^CD5^+^ B Cells in Spleen from Pristane-treated Cd38^−/−^ Mice

**Figure 3:**
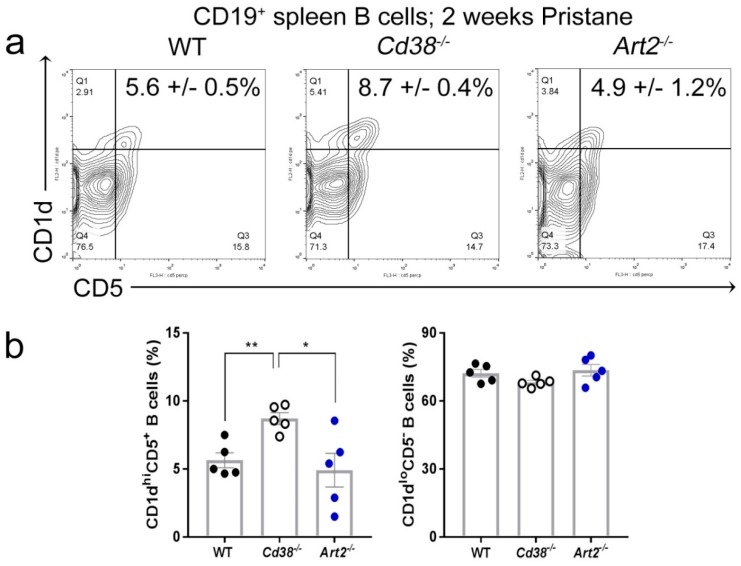
Increased frequency of CD1d^hi^CD5^+^ B cells in spleen from pristane-treated *Cd38^−/−^* mice. (**a**) Spleen cells from 2-week pristane-treated mice were stained with anti-CD19-Allophycocyanin (APC), anti-CD38-Fluorescein isothiocyanate (FITC), anti-CD5-Peridinin-chlorophyll-protein complex (PerCP), and anti-CD1d-Phycoerythrin (PE) mAbs. Contour-plots of CD1d vs. CD5 of CD19^+^-gated B cells of a representative experiment are shown. In quadrant Q2, the percentages ± SEM of CD1d^hi^CD5^+^ B cells are highlighted. (**b**) Bars represent mean ± SEM of frequencies of CD1d^hi^CD5^+^ B cells (left panel), and CD1d^lo^CD5^−^ B cells (right panel) in spleen of WT, *Cd38^−/−^*, and *Art2^−/−^* mice injected i.p. with pristane 2 weeks before. Each dot represents the value from 1 distinct mouse (*n* = 5 mice per group). * *p* = 0.0203; ** *p* = 0.0022. Unpaired *t*-test. The results are representative of at least two independent experiments.

Since reduced numbers and/or functions of iNKT cells have been shown to exacerbate pristane-induced lupus nephritis [24], we assessed whether the absence of ART2 or CD38 had any influence on the frequency of CD1d^hi^CD5^+^ B cells in the spleen of mice treated with pristane. In pristane-treated mice, the frequency of CD1d^hi^CD5^+^ B cells was significantly higher in *Cd38^−/−^* than in WT and *Art2^−/−^* mice (Figure 3). Note that B10 cells are particularly enriched within the CD1d^hi^CD5^+^ subset, where they represent 15–20% of the cells of this subset in C57BL/6 mice [25]. Together with this increased frequencies in CD1d^hi^CD5^+^ B cells, a significant increase of CD1d expression on these cells was detected in diseased *Cd38^−/−^* mice (fluorescent mean intensity (FMI) = 475 ± 10.2, *n* = 5) as compared to WT mice (FMI = 399.8 ± 7.7, *n* = 5, *p* = 0.0014) and diseased *Art2^−/−^* mice (FMI = 369.4 ± 13.1, *n* = 5, *p* < 0.0001). These results resemble the observed upregulation of CD1d on MLN B cells from mice under a chronic intestinal inflammatory condition, which produced IL-10, and suppressed progression of intestinal inflammation [26].

### 2.4. Decreased Percentages of Plasmacytoid Dendritic Cells (pDCs) and Diminished IFN-α Secretion in Peritoneal Lavage Fluids of Pristane-Treated Cd38^−/−^ Mice

**Figure 4:**
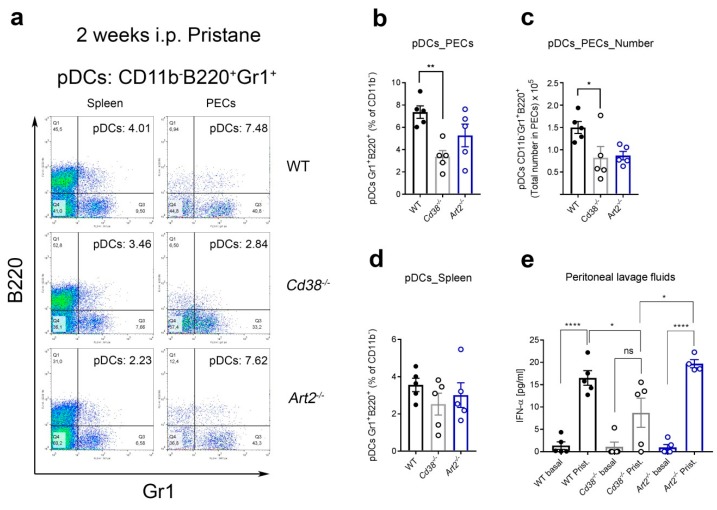
Decreased percentages of plasmacytoid dendritic cells (pDCs) and diminished IFN-α secretion in peritoneal lavage fluids of pristane-treated *Cd38^−/−^* mice. (**a**) Two weeks after pristane i.p. injection, spleen cells and peritoneal exudate cells (PECs) from WT, *Cd38^−/−^*, and *Art2^−/−^* mice were stained with anti-B220-FITC, anti-CD11b-APC, anti-Gr1-PE, and anti-CD5-PerCP mAbs. After gating the CD11b^−^ cells, pseudocolor-plots of B220-FITC vs. Gr1-PE are shown. Gating strategies for PECs pDCs and spleen pDCs are shown in Figures S2 and S3, respectively. In Q2, percentages of pDCs are highlighted in a representative experiment. According with Nakano et al [27], pDCs are CD11b^−^, and double-positive for B220 and Gr1. Pseudocolor plots display the relative population density of cell populations within the graph window along two parameters, blue and green dots correspond to areas of lower cell density, red and orange are areas of high cell density, and yellow is mid-range (**b**) Mean ± SEM of percentages of PECs pDCs from 2-week-pristane treated mice. ** *p* = 0.0064, ordinary 1-way ANOVA corrected with Tukey’s multiple comparisons test. (**c**) Mean ± SEM of pDC numbers in PECs from 2-week-pristane mice. * *p* = 0.0426. (**d**) Mean ± SEM of percentages of spleen pDCs from 2-week-pristane treated mice (*n* = 5 mice per group). (**e**) Peritoneal lavage fluids were harvested form 2week pristane-treated mice (*n* = 4–5 mice per group) as described in Material and Methods. IFN-α levels were quantified by ELISA. * *p* = 0.0473, **** *p* < 0.0001, ordinary 1-way ANOVA corrected with Bonferroni’s multiple comparisons test. The results are representative of at least three independent experiments for peritoneal pDCs and two experiments for spleen pDCs.

In the pristane lupus model, we also tested whether the frequency of pDCs and IFN-α production was compromised in *Cd38^−/−^* mice. As shown in Figure 4a,b, the frequencies of pDCs were significantly reduced in peritoneal exudate cells (PECs) from pristane-treated *Cd38^−/−^* mice. In contrast, no significant differences were observed in the frequencies of pDCs in spleen cells of these mice (Figure 4b). The low proportion of pDCs correlated with lower amounts of IFN-α detected in the peritoneal lavage fluids of the *Cd38^−/−^* mice as compared with that in WT and *Art2^−/−^* mice (Figure 4c). Indeed, these data are in agreement with our previous study where pristane-treated *Cd38^−/−^* mice showed reduced autoantibody, type I IFN signature, and kidney pathology compared to WT and *Art2^−/−^* mice [17].

### 2.5. Increased Frequencies of B10 + B10pro Cells in Spleen from Cd38^−/−^ Mice

**Figure 5:**
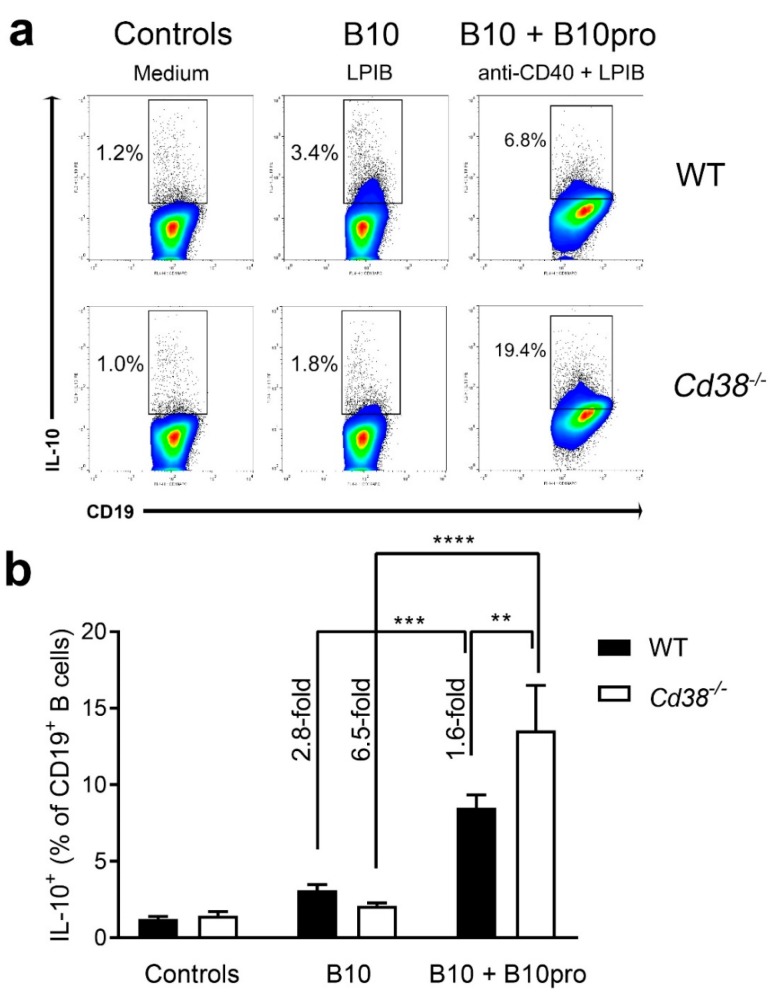
Increased frequencies of B10 + B10pro cells in spleen from *Cd38^−/−^* mice. (**a**) Spleen cells from naïve WT and *Cd38^−/−^* mice were cultured with medium alone (control cells, left panels), with LPS + PMA + Ionomycin + Brefeldin A (LPIB) for 5 h (B10 cells, middle panels), or with anti-CD40 agonistic mAb for 48 h, with LPIB added during the last 5 h of culture (B10 + B10pro cells, right panels). Then cells were stained with CD19 mAb, permeabilized, and stained with IL-10 mAb for flow cytometry analysis. Gates show the percentages of IL-10 producing cells among total CD19^+^ B cells. Pseudocolor/smoothing/outliers plots are represented. Blue and green colors correspond to areas of lower cell density, red and orange are areas of high cell density, and yellow is mid-range. Outliers represent the lowest density of cells and are represented as black dots. (**b**) Bars represent the mean ± SEM of the percentages of IL-10^+^ CD19^+^ B cells in controls, B10 cells, and B10 + B10pro cells from WT splenocytes (closed bars) or *Cd38^−/−^* splenocytes (open bars). The frequency of CD19^+^ B cells that express IL-10^+^ increases significantly after 48 h of anti-CD40 stimulation, reflecting maturation of B10pro cells into IL-10 competent B10 cells. ** *p* = 0.0051; *** *p* = 0.0003; **** *p* <0.0001. Ordinary two-way ANOVA corrected using Tukey’s multiple comparisons test. The results are representative of at least three independent experiments.

Because IL-10 production is the hallmark of B10 cells, IL-10 secretion by spleen cells was investigated following a 5 h LPIB stimulation as described in Material and Methods. As shown in Figure 5, the proportion of IL-10-expressing B cells was higher in spleen from WT than from *Cd38^−/−^* mice (3.1 ± 0.37% in WT vs. 2.09 ± 0.18% in *Cd38^−/−^*, *n* = 7 per group), although these differences were not statistically significant. Note that B10 cells are phenotypically distinct although they share some overlapping phenotypic markers with other regulatory B cells subsets, including the transitional-2 (T2)-Marginal Zone (MZ) precursor B cells [25], which are highly positive for CD38 expression in WT mice, and show decreasing frequencies in *Cd38^−/−^* splenocytes [28].

B10 progenitor (B10pro) cells have also been identified within the spleen CD1d^hi^CD5^+^CD19^hi^ B-cell subset [13]. B10 pro cells are defined as those B cells that are not induced to express cytoplasmic IL-10 after LPIB stimulation for 5 h, but that can be induced to mature into IL-10 competent B10 cells by ex vivo culture with agonistic CD40 mAb stimulation for 48 h [28]. Because there is not a specific marker for B10pro cells, and IL-10 expression is the defining marker for B10 cells, it was not possible to discriminate between B10 and B10pro cells after this 48 h ex vivo culture [29]. Therefore, we asked whether in *Cd38^−/−^* spleen cells CD40-mediated signals may induce B10pro-cell acquisition of IL-10 competence to distinct levels than in WT. As shown in Figure 5, significant increased frequencies of IL-10^+^ CD19^+^ B cells were observed upon stimulation with an agonist anti-CD40 mAb (48 h) + LPIB (5 h) in *Cd38^−/−^* than in WT splenocytes. Therefore, the CD40-mediated signaling pathways seemed to be more effective in *Cd38^−/−^* than in WT splenocytes to induce the acquisition of IL-10 competence of the B10pro subset. Since CD40 mAb stimulation does not induce B-cell clonal expansion during the 48 h culture period [10], these results also suggest that in the absence of CD38 the frequency of B10pro cells might be higher than in WT mice, and CD40 agonist stimulation underscores it. The contribution of IL-21 to the expansion of IL-10-producing B10 cells cannot be discarded [10], since unpurified splenocytes containing T cells were used in this assay, although we have not tested this possibility.

## 3. Discussion

The major finding of our study is that in naïve mice the frequencies of CD5^+^CD1d^hi^CD19^+^ B cells were similar in *Cd38^−/−^* and WT mice, while upon in vivo treatment with pristane, the proportion of these cells augmented significantly in *Cd38^−/−^* mice. Since B10 cells, which strongly controls T cell-mediated inflammatory responses, are highly enriched within the CD5^+^CD1d^hi^CD19^+^ B cell subset [12], this data may reflect increased frequency and number of B10 cells in pristane-treated *Cd38^−/−^* mice, which further suggests better in vivo capabilities to expand IL-10-producing B cells in the absence of CD38. The functional ex vivo assays showing increased frequencies of IL-10-producing B cells in *Cd38^−/−^* splenocytes than in WT upon stimulation with an agonist anti-CD40 mAb are in agreement with the in vivo experiments in the pristane-induced lupus model. Since this does not occur under steady-state conditions (naïve mice), or short-term pharmacological stimulation (LPS + PMA + Ionomycin), these results strongly suggest that *Cd38^−/−^* mice are better suited than WT mice to generate regulatory B10 cells under strong inflammatory conditions, or under the appropriate agonist stimulation.

Another major finding in this study is based on the observation that naïve CD38-deficient mice show similar frequencies of IL-10-producing B cells upon treatment of spleen cells with LPIB treatment, which functionally defines the so-called B10 cells. Our results confirm and extend previous data obtained by Domínguez-Pantoja and colleagues, which showed decreased proportion of CD5^+^CD1d^hi^CD19^+^ B cells in naïve *Cd38^−/−^* mice, whereas the proportion of IL-10-producing cells within this B cell subset was similar to that in WT [23]. It is interesting to note that these authors tested the frequencies of CD5^+^CD1d^hi^CD19^+^ B cells upon 48 h in vitro culture with either medium alone or in the presence of LPS (48 h) and PIM (5 h), while we tested that in freshly isolated spleen cells from naïve mice or pristane-treated mice. Secondly, our functional data on IL-10 production were obtained in cells that were stimulated with LPS for short periods of time (5 h), after treatment with an anti-CD40 mAb (48 h in total), or without anti-CD40 pre-treatment, while their procedure always involved LPS stimulation for 48 h. It is known that the latter treatment induces strong proliferation of B10pro and B10 cells, while anti-CD40 stimulation induces maturation of B10pro cells into IL-10 competent B10 cells, with minimal effect on proliferation and expansion of these cells [10]. Therefore, our method allowed us to determine both B10 cell and B10pro + B10 cell numbers individually, and to observe important differences in B10 cell frequencies relative to B10pro + B10 cell frequencies, while theirs did not. Therefore, differences in the methodological approaches may lead to divergent results. Thus, we report that 48 h treatment of spleen cells with a CD40 agonistic mAb induces a stronger IL-10 response in *Cd38^−/−^* than in WT. Since this treatment induces the maturation of B10pro to B10 cells without inducing cell proliferation, these results strongly suggest that in spleen cells from *Cd38^−/−^* mice the proportion of B10pro is higher than that in WT mice. Alternatively, we cannot discard that *Cd38^−/−^* spleen B cells respond better to CD40 engagement due to increased signaling capabilities via IL-21 production by T cells and CD40-CD40L interactions of B10 cells with T cells as compared with WT counterparts. The increased capability of CD40 engagement to induce the maturation of B10pro into IL-10 producing B cells in *Cd38^−/−^* mice is indeed in agreement with the concept that B10-cell maturation into functional IL-10-secreting effector cells requires CD40-dependent cognate interactions with T cells [10].

**Figure 6:**
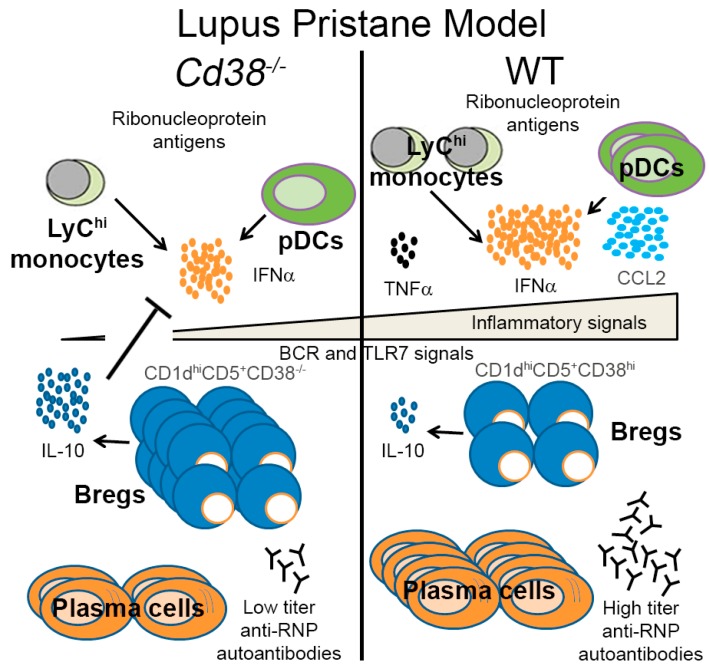
Model for pristane-induced lupus. This model is a modification of that proposed by Menon et al. [30], for human SLE, and it is based on our own previous results on the pristane-induced lupus model [17], and on the results shown in the current study.

One possible explanation for the results of increased frequencies of CD5^+^CD1d^hi^CD19^+^ B cells in the context of the pristane lupus model has to do with the low proportion and numbers of peritoneal pDCs, and the decreased levels of IFN-α in *Cd38^−/−^* mice 2 weeks after pristane treatment (Figure 4). Our working hypothesis is that in pristane-treated *Cd38^−/−^* mice the low number of pDCs, and decreased production of IFN-α, in a relatively mild inflammatory environment, may facilitate the maturation of B10pro into IL-10-producing B10 cells. The increased production of IL-10 may help to further decrease the IFN-α release. Although we do not know at this point whether this mechanism operates in the pristane-induced lupus model, there is strong indirect evidence that this could be the case. Thus, it is interesting to note that in healthy individuals, human pDCs drive the differentiation of CD19^+^CD24^hi^CD38^hi^ (immature) B cells into IL-10-producing CD24^+^CD38^hi^ Breg cells and plasmablasts, via the release of IFN-α and CD40 engagement. CD24^+^CD38^hi^ Breg cells conversely restrain IFN-α production by pDCs via IL-10 release. In contrast, in SLE, this cross-talk is compromised; pDCs promote plasmablast differentiation but fail to induce Breg cells [30]. According to the authors this is due in part to aberrant production of IFN-α in a strong inflammatory environment [30]. Whether this mechanism is fully operative in lupus mouse models requires further investigation, however it is interesting to note that early, transient depletion of pDCs ameliorates autoimmunity in a lupus model [31]. Indeed, we have shown in the pristane-induced lupus model that in *Cd38^−/−^* mice there is reduced autoantibody production, type I IFN signature, and inflammatory signature compared to WT and *Art2^−/−^* mice [17]. According to the model proposed by Menon et al. [30], the relatively low IFN-α levels observed in *Cd38^−/−^* mice (Figure 4e) should be enough to help differentiate immature B10pro cells into IL-10-producing B10 cells in a relatively mild inflammatory environment. In contrast, WT and *Art2^−/−^* mice produce more IFN-α and are subjected to a stronger inflammatory environment than *Cd38^−/−^* mice, so this process might be less efficient, hence the lower frequency of pDCs observed. In light of these results, we propose a working model in Figure 6, which may facilitate future research into this subject. In this sense, sorting of the CD1d^hi^CD5^+^CD38^hi^CD19^+^ subset and adoptive transfer experiments would provide further insights on the role of regulatory B10 cells in the pristane lupus model. Alternatively, the ex vivo expansion and reinfusion of autologous B10 cells strategy, which has been successfully used in other autoimmune diseases [10], may provide an effective in vivo treatment for this mouse model of SLE.

## 4. Materials and Methods

### 4.1. Mice

*Cd38*^−/−^ and *Art2^−/−^* mice [32,33] were backcrossed for 12 generations to the C57BL/6 J (B6) background, bred, and maintained under specific pathogen-free conditions at the IPBLN-CSIC Animal Facility (register No: ES-180210000022) in Granada, Spain until they were 4 weeks-old. Wild-type C57BL/6 J mice were purchased from Charles River (Barcelona, Spain). 8–12-week-old mice received a single dose of 0.5 mL pristane (2,6,10,14-tetramethylpentadecane [34]; Sigma-Aldrich, Merck KGaA, Darmstadt, Germany) that was filtered through a 0.25-μm filter and administered intraperitoneally (i.p.). Control mice received either a single dose of 0.5 mL saline or were left untreated. Peritoneal cells, spleen, and blood were harvested 2 weeks after treatment. All experimental procedures involving animals at IPBLN-CSIC were approved by the Institutional Animal Care and Use Committee (Proyect SAF2011-27261, date: 14 December 2011; Project SAF-2017-89801-R, date: 24 November 2017), which follows the ARRIVE guidelines [35] in accordance with the U.K. Animals (Scientific Procedures, Act, 1986) and associated guidelines (EU Directive 2010/63/EU for animal experiments), and with the National Institutes of Health guide for the care and use of Laboratory animals (NIH Publications No. 8023, revised 1978).

### 4.2. Splenocytes

Spleens were obtained and placed into 3 mL of PBS/BSA/EDTA buffer (PBS, pH 7.2; 0.5% bovine serum albumin (BSA from Sigma-Aldrich) and 2 mM EDTA (Sigma-Aldrich)) after being weighed. The spleens were individually placed in a small petri dish between two filter papers and crushed with the end of a syringe. The cells were then filtered through a Pre-Separation filter placed on a 15 mL tube. 1 mL of PBS/BSA/EDTA buffer was used to wash the small petri dish and then extracted from the petri dish to wash the Pre-Separation filter. After adding enough buffer to each tube to reach a total volume of 10 mL, the samples were centrifuged at 1500 rpm for 5 min at 4 °C. After the supernatants were removed, 1 mL of ammonium chloride solution (STEMCELL Technologies, Grenoble, France) was used to lyse the red blood cells. The cells were then filtered through separate Pre-Separation filters again and the Pre-Separation filters were washed with 3 mL of ammonium chloride. The cell samples were incubated in ice for 10 min. After adding 8 mL of PBS/BSA/EDTA buffer, the cell samples were centrifuged again at 1500 rpm for 5 min at 4 °C and the supernatants were removed. The cell samples were resuspended in 1 mL of the PBS/BSA/EDTA buffer and 4 more mL of the buffer were added.

### 4.3. Counting

First, a 1:20 dilution was used to count each cell sample. Twenty microliters of the 1:10 diluted cell sample and 20 μL of Trypan Blue was obtained in a micropipette and used. Ten microliters of the total dilution was put on the plate used for counting (Neubauer hematocytometer; BRAND GMBH + CO KG, Wertheim, Germany). The live and dead cells were counted. Calculations were made and the appropriate number of cells was obtained from each cell sample.

### 4.4. Flow Cytometry

#### 4.4.1. B Cell Subsets and pDCs

In order to analyze distinct B cell subpopulations from each of the samples, several fluorochrome-conjugated mAb mixtures were used per experiment. For regulatory B10 cells the mAb combination was the following: CD19-APC (clone 1D3), CD38-FITC (clone 90/CD38), CD5-PerCP (clone 53-7.3), CD1d-PE (clone 1B1) (all from BD Biosciences, Beckton Dickinson, Franklin Lakes, NJ, USA). For pDCs: B220-FITC (clone RA3-6B2, Biolegend, San Diego, CA, USA), CD11b-APC (clone M1/70.15.11.5, Miltenyi Biotec, Bergisch Gladbach, Germany), Gr1-PE (clone RB6-8C5, BD Biosciences), CD5-PerCP (clone 53-7.3, BD BioSciences). After adding the respective volumes of cells to two different 5 mL polystyrene (PS) tubes (Falcon, Corning, Corning, NY, USA), 2 mL of PBS/BSA/EDTA buffer was added to each tube. The cells were centrifuged at 1500 rpm for 5 min at 4 °C and then the supernatants were removed. The cells were resuspended in PBS/BSA/EDTA buffer and Mouse BD Fc Block (Rat anti-mouse CD16/CD32, clone 2.4G2, BD Biosciences) was added to both samples. The cells incubated in ice for 20 min. The samples in the PS tubes were separated into appropriate labeled tubes in order to add antibodies. 100 μL of PBS/BSA/EDTA was added to the new cell separations. The antibodies were added and incubated in ice in the dark for 20 min. 2 mL of PBS/BSA/EDTA buffer was added and centrifuged at 1500 rpm for 5 min at 4 °C. The supernatants were removed. The cells were resuspended in 100 μL of PBS/BSA/EDTA buffer and 100 μL of paraformaldehyde was added to each tube in order to fix the cells for staining.

#### 4.4.2. Intracellular IL-10 Staining

In order to analyze the proportion of IL-10-producing B cells, spleen cells were surface stained with an anti-CD19-APC (clone 1D3, BD Biosciences), following by permeabilization, fixation, and intracellular staining with an anti-IL-10-PE mAb (clone JES5-16E3, Miltenyi Biotec), as previously described [17] using the Inside Stain Kit from Miltenyi (reference 130-090-477). Briefly, after adding the respective volumes of cells to seven (eight when using CD40 for IL-10pro stimulation) different 5 mL PS tubes, 2 mL of PBS/BSA/EDTA buffer was added to each tube. The cells were centrifuged at 1500 rpm for 5 min at 4 °C and then the supernatants were removed. The cells were resuspended in PBS/BSA/EDTA buffer and centrifuged twice more. Fc Block (CD16/CD32 from BD Biosciences) was added to all the samples. The cells incubated in ice for 20 min with foil. 100 μL of PBS/BSA/EDTA was added to the samples. The antibody CD19-APC was added to the respective tubes and incubated in ice in the dark for 20 min. Two microliters of PBS/BSA/EDTA buffer was added and centrifuged at 1500 rpm for 5 min at 4 °C. The supernatants were removed. The cells were resuspended in 100 μL of PBS/BSA/EDTA buffer and centrifuged again. Two-hundred-and-fifty microliters of PBS/BSA/EDTA buffer and 250 μL of Inside Fix were added to the tubes and incubated at room temperature in the dark for 20 min. The samples were centrifuged at 1500 rpm for 5 min at 4 °C and the supernatant was removed. The samples were resuspended in 1 mL of PBS/BSA/EDTA buffer, centrifuged at 1500 rpm for 5 min at 4 °C and the supernatant was removed. One-hundred microliters of Inside Perm was added to all the cells and 0.5 μL of IL-10-PE was added to the respective tubes and incubated at room temperature for ten minutes in the dark. 1 mL of Inside Perm was added to the samples, centrifuged at 1500 rpm for 5 min at 4 °C and the supernatant was removed. The samples were resuspended in 200 μL of PBS/BSA/EDTA buffer.

#### 4.4.3. Data Acquisition and Analysis

For analysis purposes, 50,000 (150,000 for functional analysis) events were acquired from each sample using a FACSCalibur flow cytometer (BD Biosciences). The software used for data acquisition was BD CellQuest Pro (version 0.7.df2b) and further analysis was done using the FlowJo software (version 7.6.4, Tree Star Inc., Ashland, OR, USA). All of the data from the samples were acquired less than 24 h from the time the samples were ready for analysis.

### 4.5. Functional Analysis of Regulatory B (B10) Cells

B10 cells are a functionally defined subset currently identified only by their competency to produce and secrete IL-10 following appropriate stimulation. Therefore, the procedure described by Matsushita and Tedder was used [29]. Briefly, splenocytes were cultured at 37 °C in medium alone (as a control) containing RPMI 1640, FBS, penicillin, streptomycin, l-Glutamine, and 2-mercaptoethanol (all reagents from GIBCO, Thermo Fisher Scientific, Waltham, MA, USA) or in culture medium containing PMA (Sigma-Aldrich, 50 ng/mL, final concentration), ionomycin (Sigma-Aldrich, 500 ng/mL final), LPS (Sigma-Aldrich, 10 μg/mL final), to induce IL-10 production. After 1 h incubation, Brefeldin A (Sigma-Aldrich, 2 μg/mL final) was added to the sample with PMA, ionomycin, and LPS and cells were incubated for another 4 h at 37 °C. Then cells were stained for cell surface CD19 expression and cytokine capture to visualize IL-10 secreting cells as described above. For analysis of B10pro and B10 cells, splenocytes were incubated in culture medium alone (as a control), or culture medium containing the anti-CD40 mAb (Clone FGK45.5, pure-functional grade, Miltenyi, Cat No. 130-093-022, 1 μg/mL, final) for 43 h at 37 °C and then another 5 h with the LPIB cocktail described above.

### 4.6. IFN-α ELISA

All 14 mouse IFN-α subtypes were measured using the Verikine Mouse Interferon-alpha ELISA (PBL Assay Science, Piscataway, NJ, USA). Assays were performed according to the manufacturer’s protocol.

### 4.7. Statistical Analyses

Statistical analyses were performed using the GraphPad Prism 7 software (GraphPad Software, San Diego, CA, USA).

## Supplementary Materials

Supplementary materials can be found at http://www.mdpi.com/1422-0067/19/10/2906/s1.

## Abbreviations

Art2	T-cell ecto-ADP-ribosyltransferase 2
B10	IL-10-producing B cells
B10pro	B10 progenitor cells
Bregs	Regulatory B cells
LPIB	LPS + PMA + Ionomycin + Brefeldin A
LPS	Lipopolysaccharide
pDCs	Plasmacytoid Dendritic Cells
PECs	Peritoneal Exudate Cells
PIM	PMA + Ionomycin + Monensin
PMA	Phorbol 12-Myristate 13-Acetate
Pristane	2,6,10,14-Tetramethylpentadecane
SLE	Systemic Lupus Erythematosus
TRPM2	Transient Receptor Potential Melastatin 2
WT	Wild-Type
