# Supplementary material for: The Role of CD38 on the Function of Regulatory B Cells in a Murine Model of Lupus
Source: Int J Mol Sci. 2018 Sep 25;19(10):2906. doi: 10.3390/ijms19102906 (PMC6213330; doi:10.3390/ijms19102906)
Supplement: Supplementary file 1 [file ijms-19-02906-s001.pdf]

Supplementary Materials

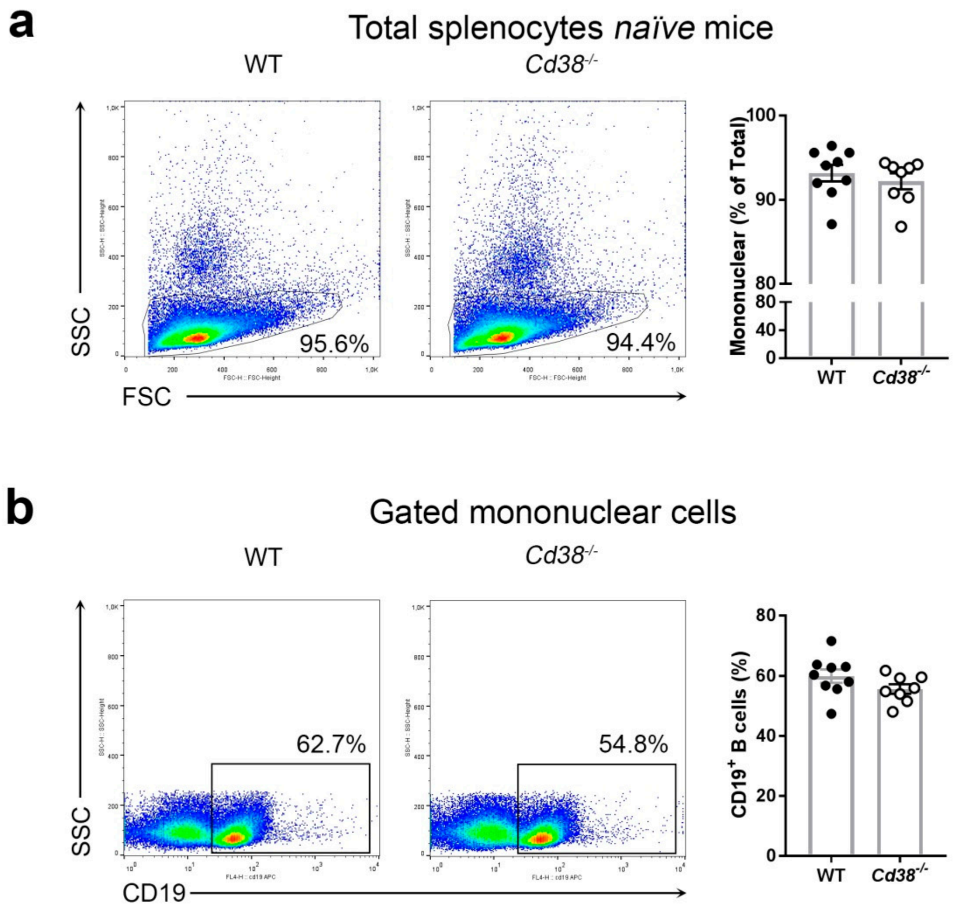

Figure S1. Related to Figure 1. Gating strategy for immunophenotyping splenocyte B cells from naïve *Cd38*$^{-/-}$ and WT mice before showing CD1d^{hi}CD5^{+} B cell frequencies in Figure 1. Percentages of mononuclear and total B cells are shown.

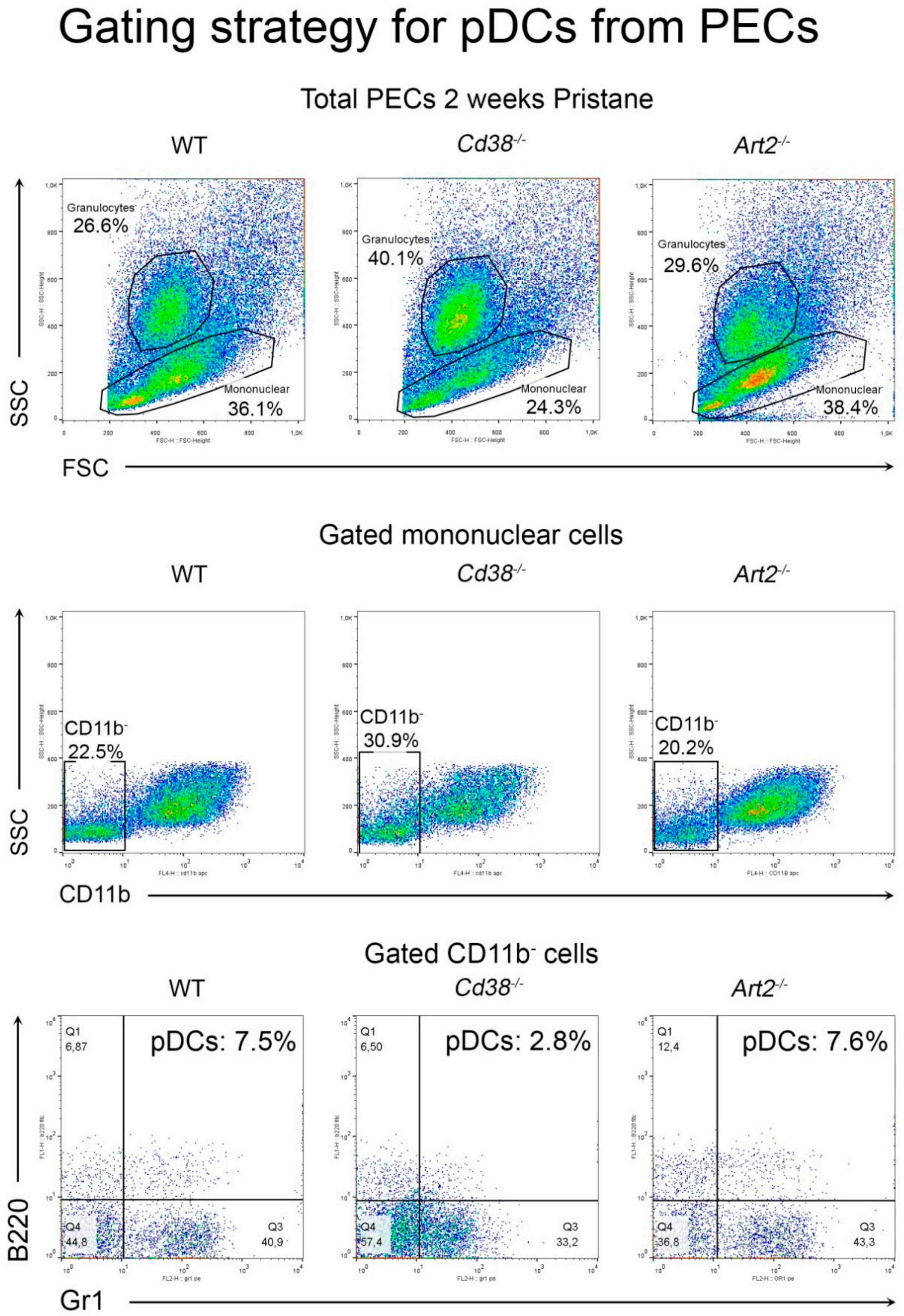

Figure S2. Related to Figure 4. Gating strategy for immunophenotyping pDCs from PECs of mice injected with pristane 2 weeks before. pDCs correspond to the double positive cells for Gr1 and B220 of the gated CD11b$^-$ subpopulation.

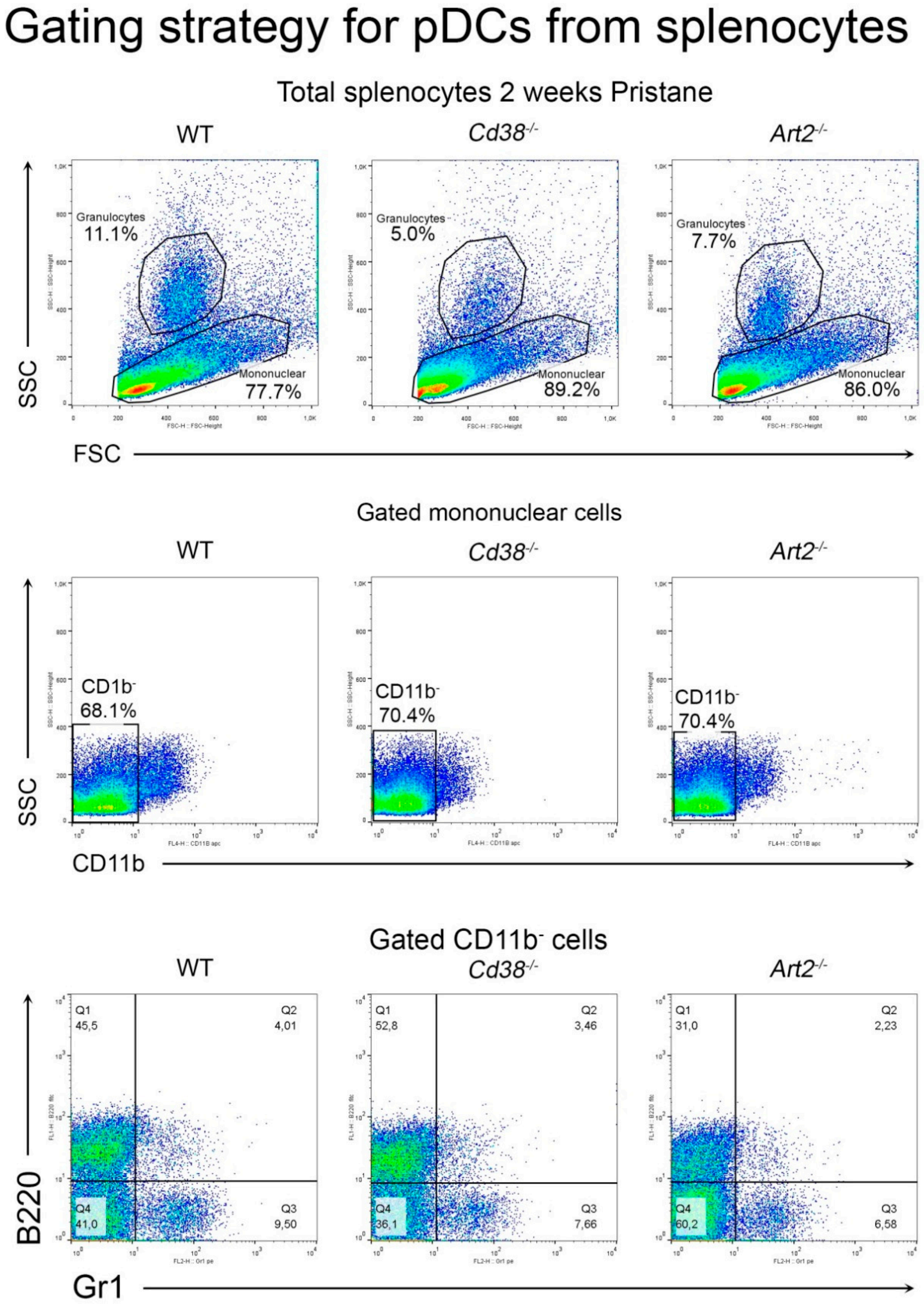

Figure S3. Related to Figure 4. Gating strategy for immunophenotyping pDCs from splenocytes of mice injected with pristane 2 weeks before. pDCs correspond to the double positive cells for Gr1 and B220 of the gated CD11b- subpopulation.
